# Continuity of care for patients with dementia during COVID-19 pandemic: flexibility and integration between in-person and remote visits

**DOI:** 10.3389/fpubh.2023.1301949

**Published:** 2024-01-08

**Authors:** Daniele Emedoli, Elise Houdayer, Pasquale Anthony Della Rosa, Alice Zito, Luigia Brugliera, Paolo Cimino, Jeffrey David Padul, Andrea Tettamanti, Sandro Iannaccone, Federica Alemanno

**Affiliations:** ^1^Department of Rehabilitation and Functional Recovery, IRCCS San Raffaele Scientific Institute, Milan, Italy; ^2^Department of Neuroradiology, IRCCS San Raffaele Scientific Institute, Milan, Italy

**Keywords:** telemedicine, telerehabilitation, cognitive disorders, cognitive training, neuropsychology, COVID-19, continuity of care

## Abstract

**Introduction:**

During the pandemic, the Cognitive Disorders Unit of San Raffaele Hospital (Milan, Italy) offered patients the opportunity to undergo neuropsychological evaluations and cognitive training through telemedicine.

**Method:**

We conducted an investigation to assess how patients responded to this option and to determine if telemedicine could ensure continuity of care.

**Results:**

Between October 2019 and May 2022, a total of 5,768 telemedicine appointments and 8,190 in-person outpatient appointments were conducted, resulting in an increase in the rate of telemedicine activity from 16.81% in January 2020 to 23.21% in May 2022. Peaks in telemedicine activity reached 85.64% in May 2020 and 83.65% in February 2021, both representing a significant portion of the total activity. Interestingly, there was a notable positive correlation between telemedicine activity and the worsening of the Italian pandemic (*r* = 0.433, *p* = 0.027).

**Discussion:**

During the peaks of contagion, the total number of visits remained stable, highlighting that telemedicine effectively served as a valuable and efficient tool to ensure continuity of care for vulnerable patients. This was evident from the integration of remote visits with in-person appointments.

## Introduction

During the COVID-19 pandemic, issues related to discontinuing care have arisen due to hospitals needing to close numerous clinical departments and allocate most beds to COVID-19 patients. Furthermore, because of the emergency, many outpatient clinics were either closed for months or limited their services to emergencies only. To address these interruptions, the use of telemedicine was expanded, alongside digital solutions and advanced technology interfaces (1, 2). Telemedicine, along with tools rooted in artificial intelligence, big data analytics, and mobile tracing apps for surveillance, was extensively utilized globally for diagnosing, preventing, monitoring, and treating individuals (3, 4). Concomitantly with the drastic reduction in outpatients appointments, there has been a surge in the utilization of remote consultations (5). Patients, caregivers and clinicians rated the use of telemedicine during the recent pandemic as highly satisfactory (6). Consequently, the majority of patients and healthcare providers expressed a willingness to continue using telemedicine even after the pandemic (7, 8).

In 2019, before the onset of the COVID-19 pandemic, the Cognitive Disorders Unit at San Raffaele Hospital (Milan, Italy) had established telemedicine services for conducting remote neuropsychological evaluations and cognitive training. This service was offered to patients already undergoing treatment at our Institute, primarily those receiving care for dementia and acquired brain injuries. Dementia currently stands as the seventh leading cause of death worldwide and significantly contributes to disability and dependency among older populations. The statistics regarding dementia and its impact on patients, their families, and the entire healthcare system are staggering (9). Evidence reveal escalating pressures on families and caregivers, both emotionally and financially, alongside substantial financial strains on the entire healthcare system (10). Hence, telemedicine services represent an innovative solution that lessens the burden on patients’ support networks, is more environmentally friendly, and incurs reduced costs compared to in-person appointments (11–13). While further investigations are necessary to gauge the environmental impact and social costs, telemedicine has been reported as a feasible approach to assist individuals with dementia stay connected to their service providers amid the pandemic (14). The usability and efficacy of teleneuropsychology assessments and training have already been investigated (15–18). Data reported in the litterature showed good evidence for the validity of teleneuropsychology assessments in older adults and an efficacy of telecognitive rehabilitation at least as strong as face-to-face cognitive training. Additionally, prior studies have already indicated that both patients and clinicians found teleneuropsychology services satisfactory during the COVID-19 pandemic (8, 19). It is now accepted that telemedicine is useful but not sufficient and should not replace in-patient services but should complement traditional visits (7). More information should be collected regarding the integration of teleneuropsychology services into traditional care and how patients might respond to such offerings, especially in challenging situations like during a pandemic.

In this study, our primary objectives were to assess patients’ receptiveness to telemedicine services provided by the Cognitive Disorders Unit during the COVID-19 pandemic, investigate potential disruptions in continuity of care due to the various pandemic waves, and determine patients’ preferences continued telemedicine use versus returning to in-person outpatient care. This was particularly considered in light of the pandemic’s reduced impact in Italy since spring 2022.

## Materials and methods

### Telemedicine and outpatients’ clinic activity

We performed a retrospective analysis of telemedicine and outpatient clinic appointments administered by the Cognitive Disorders Unit at the San Raffaele Scientific Institute in Milan, Italy, between October 1st, 2019, and May 30th, 2022, as our primary outcome. Throughout this entire period, patients were given the option to receive care either in the outpatient clinic or through telemedicine. Despite the closure of numerous outpatient clinics and the reduced activity of others within our Institute, the Neuropsychology Service remained accessible to patients throughout the pandemic. The only prerequisite for patients to qualify for telemedicine was that they had previously undergone at least one neurological visit and one neuropsychological evaluation in the outpatient clinic. The telemedicine and outpatient clinic appointments considered in this study encompassed neuropsychological evaluations for patients’ follow-up and cognitive training sessions.

Telemedicine appointments were conducted remotely using video-conferencing software provided by our Institute, ensuring utmost confidentiality and privacy in a designated private room. Notably, patients who had tested positive for SARS-CoV-2 within the last 14 days were temporarily restricted from accessing the outpatient clinic during this period to mitigate the risk of contagion. The study was approved by the local Ethics committee of the San Raffaele Hospital (protocol number: PROTECT-COVID).

### Correlations with the pandemic severity

As a secondary outcome, we correlated the severity of the Italian pandemic situation, extracted from the number of symptomatic patients hospitalized with SARS-CoV-2 in Italy from February 2020 to May 2022 (20), with the numbers of telemedicine and outpatients’ clinic appointments.

### Statistical analyzes

First, we calculated the monthly percentage change (MPC) for both the outpatients and telemedicine activity time series with respective bootstrapped confidence interval (CI 95%) to explore the temporal trend of the two time series during the COVID-19 pandemic (February 2020 to May 2022).

Second, to test whether observed variations in the number of cases in the telemedicine activity series and the number of cases outpatient activity series shared any temporal association, a cross-spectral analysis was performed. A bivariate model was fitted to the time series during the COVID-19 pandemic (February 2020 to May 2022) to quantify the frequency-related squared coherence (i.e., the strength of dependency between the two time series at a particular period) and phase shift between in person medicine (the independent variable) and telemedicine (the dependent variable). The spectral estimates were smoothed with a Hamming window of width 5.

Third, a bivariate correlation analysis with bootstrapping (*n* = 5,000 samples) was run to assess the relationships between the volume of telemedicine appointments and the severity of the pandemic,. To ensure the validity of our analyzes, we also performed analyzes of variance with the Levene Test for Equality of Variances. Statistical significance was determined at a threshold of *p* < 0.05. All data analyzes were carried out using the commercially available IBM SPSS Statistics version 23 (IBM Corp. ^©^) software.

## Results

Between October 1st, 2019, and May 30th, 2022, 225 patients benefited from neuropsychological evaluations or cognitive training and were included in the analyzes (103 Female, mean age 71.53 ± 15.36 years). The patient’s population consisted in post-traumatic disorders (8%), post-stroke patients (23%) and dementia patients (69%) (Alzheimer’s disease, Mild Cognitive Impairment, Frontotemporal Dementia, Lewy Body Dementia).

A total of 13,958 treatments were included in the analyzes: 2,512 neuropsychological evaluations (18% of the total treatments) and 11,446 cognitive training (82% of the total treatments). Among these 13,958 treatments, 5,768 appointments were conducted in telemedicine and 8,190 in-person appointments were conducted in the outpatients’ clinic.

During this period, the rate of telemedicine activity increased from 16.81% in January 2020 to 23.21% in May 2022. Peaks in telemedicine activity reached 85.64% in May 2020 and 83.65% in February 2021 (Table 1).

**Table 1:** Telemedicine and outpatients’ clinic activity, over time.

	Telemedicine		Outpatients clinic	
	Number of visits	Percentage of total activity (%)	Number of visits	Percentage of total activity (%)
October ‘19	40	8.47	432	91.53
November ‘19	60	11.67	454	88.33
December ‘19	80	17.13	467	82.87
January ‘20	80	16.81	396	83.19
February ‘20	160	24.92	482	75.08
March ‘20	161	38.80	254	61.20
April ‘20	201	72.04	78	27.96
May ‘20	310	85.64	52	14.36
June ‘20	183	74.09	64	25.91
July ‘20	123	64.40	68	35.60
September ‘20	128	33.16	258	66.84
October ‘20	132	29.01	323	70.99
November ‘20	168	42.00	232	58.00
December ‘20	270	71.05	110	28.95
January ‘21	302	74.02	106	25.98
February ‘21	440	83.65	86	16.35
March ‘21	280	76.09	88	23.91
April ‘21	228	71.47	91	28.53
May ‘21	192	65.31	102	34.69
June ‘21	242	48.21	260	51.79
July ‘21	122	27.60	320	72.40
September ‘21	142	33.18	286	66.82
October ‘21	166	34.87	310	65.13
November ‘21	263	52.29	240	47.71
December ‘21	286	48.31	306	51.69
January ‘22	181	30.83	406	69.17
February ‘22	206	33.17	415	66.83
March ‘22	180	29.80	424	70.20
April ‘22	186	29.15	452	70.85

**Figure 1:**
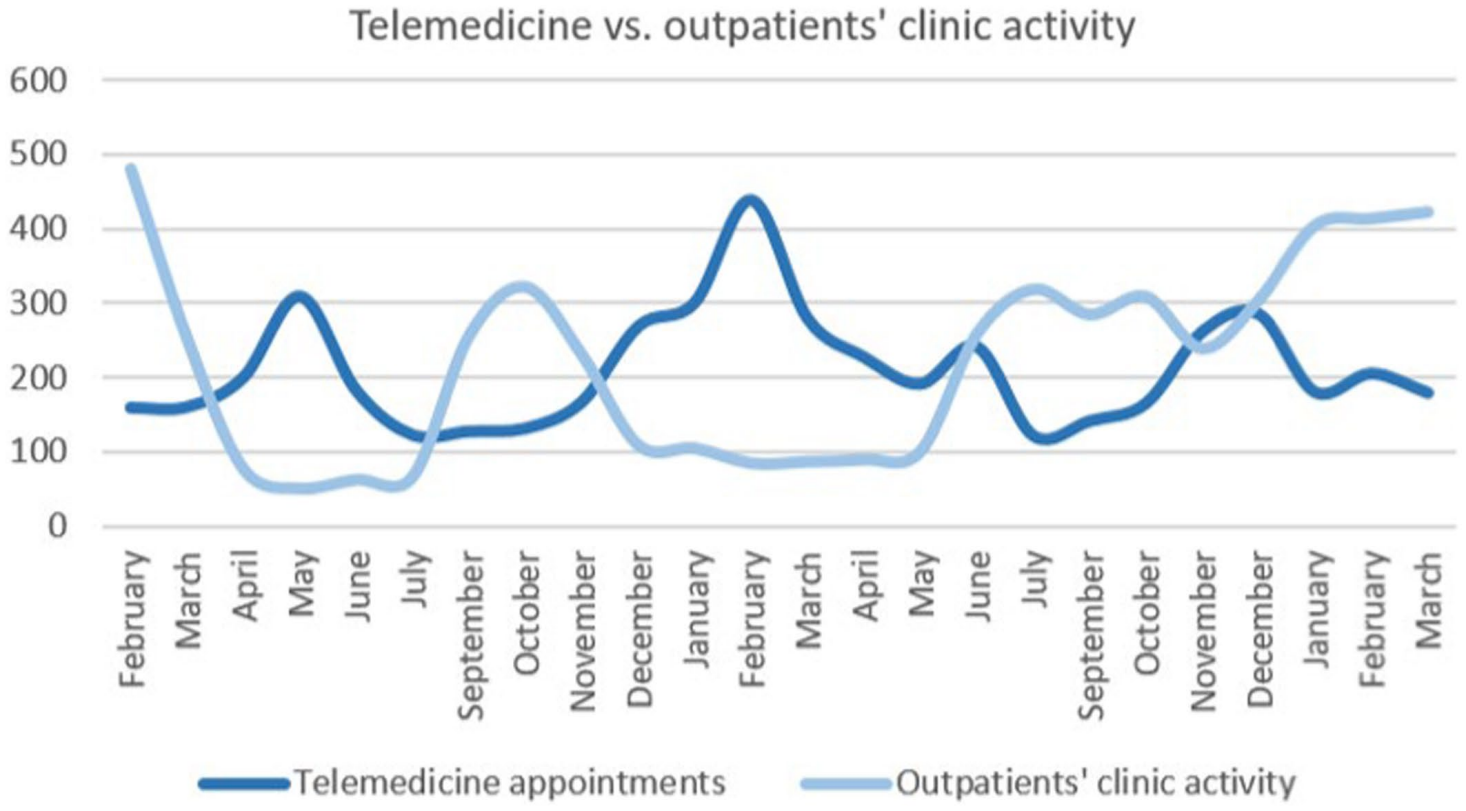
Telemedicine and outpatients’ clinic activity. It shows the evolution of telemedicine appointments (dark blue) and outpatients’ clinic activity (light blue) during the COVID-19 pandemic (February 2020 to May 2022).

In-person appointments in the outpatients’ clinic had a greater variability compared to telemedicine appointments, especially during the year 2020, as shown by the standard deviations analyzes (in-person appointments sd = 143.13 vs. telemedicine sd = 76.30, *p* = 0.017) (Figure 1).

The monthly percentage change index for the outpatients (mean percentage variation = 13.83; 95% CI: −8.58, 42.67) and telemedicine activity series (mean percentage variation = 5.14%; 95% CI: −6.43, 16.46) during the COVID-19 pandemic, shows a positive trend concerning telemedicine continuity and utility.

**Figure 2:**
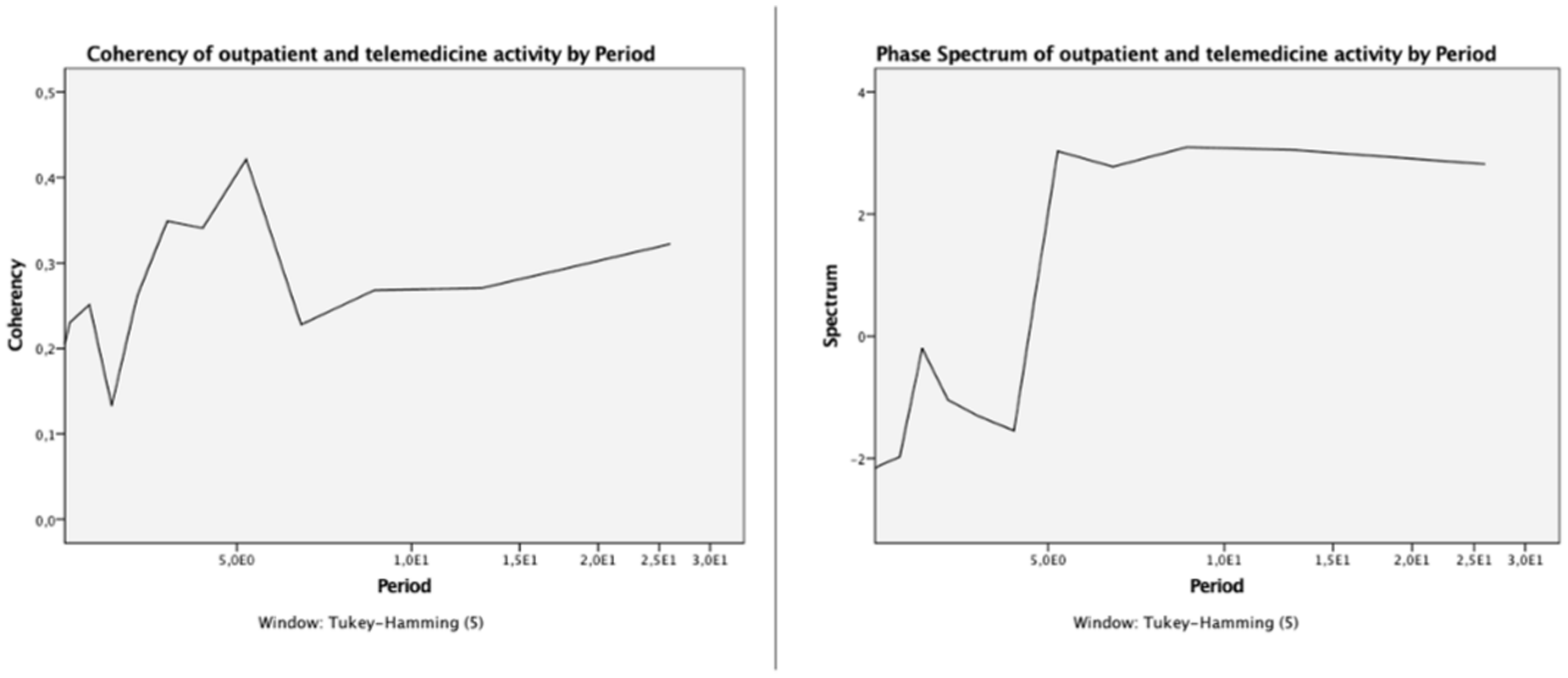
Coherency spectrum (left panel) and Phase Spectrum of outpatient and telemedicine activity during the COVID-19 pandemic (February 2020 to May 2022).

The bivariate spectral analysis yielded a significant common movement in the two series, with a significant peak involving a squared coherence of 0.421 (*p* = 0.032; phase angle, 3.03 radian) (see Figure 2), corresponding to a Fourier period of 5.2 months and with a 2.5-month lead relationship between the two time series.

**Figure 3:**
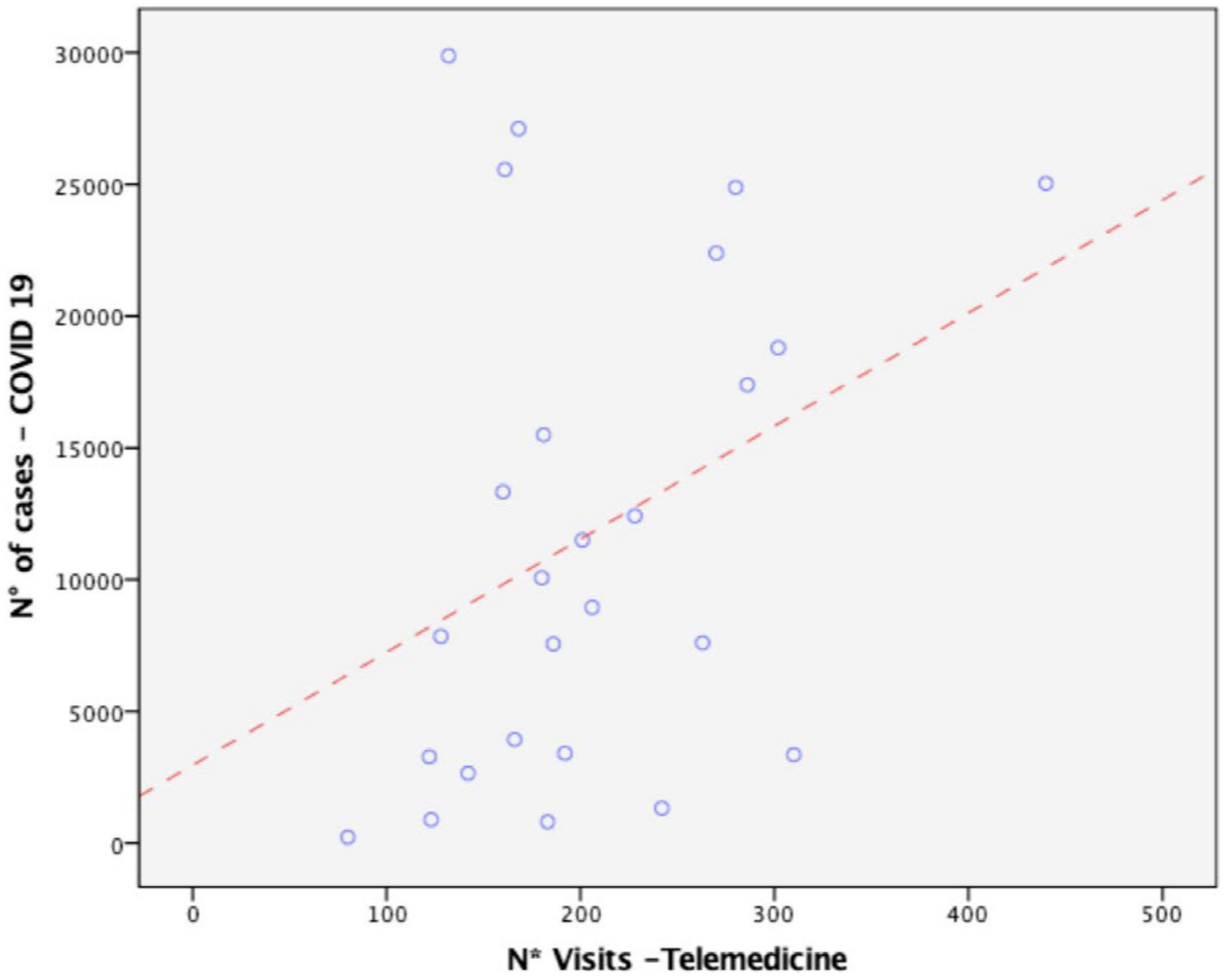
Positive association between telemedicine activity and the severity of the COVID-19 pandemic in Italy.

There was a significant positive correlation (*r* = 0.354, *p* = 0.038) between the number of telemedicine appointments and pandemic worsening expressed as the number of symptomatic patients hospitalized with SARS-CoV-2 in Italy (Figure 3).

**Figure 4:**
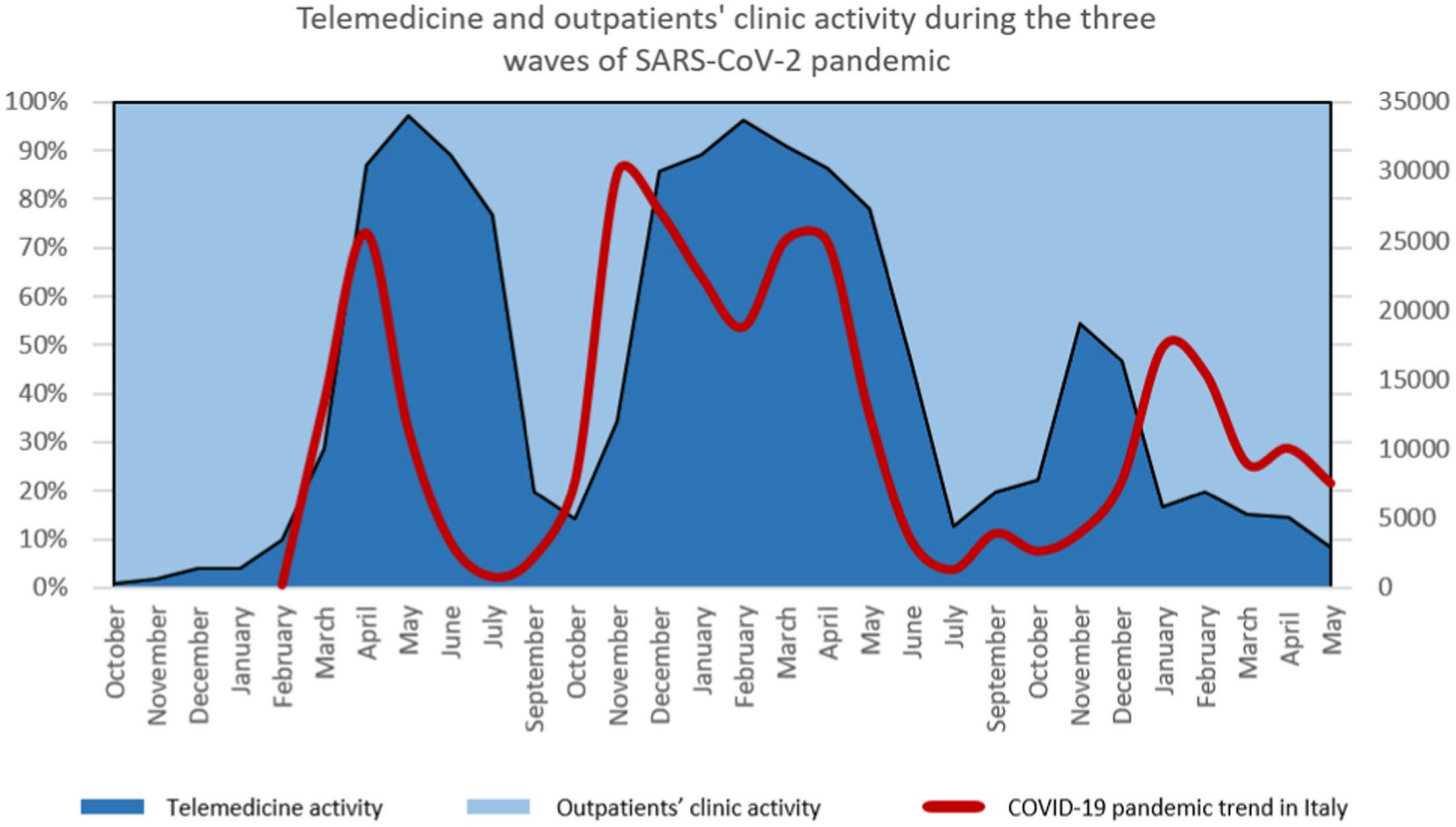
Telemedicine activity and pandemic trend in Italy. Percentages of telemedicine appointments (dark blue) and percentages of outpatients’ clinic appointments (light blue) are expressed and superimposed with the pandemic trend in Italy (red line), expressed as the numbers of hospitalized COVID-19 patients with symptoms over the national Italian territory.

The first two peaks of SARS-CoV-2 contagion (March 2020 and November 2021) were immediately followed by an increase in telemedicine appointments. Strikingly, patients were faster to switch from in-person to telemedicine appointments at the third worsening of the Italian pandemic situation. This was reflected by the rise in telemedicine activity preceding the third peak of the pandemic, in Italy (Figure 4).

## Discussion

In recent years, teleneuropsychology has been recognized as a valid tool for patients’ assessments (16, 21, 22) and cognitive training (17, 18). we demonstrated how patients with dementia transitioned between modalities, moving from face-to-face visits to telemedicine and back to face-to-face appointments, depending on the severity of the pandemic situation. This suggests that telemedicine has the potential to become an integrated component of clinical practice for neuropsychology services. Our results showed that patients preferred telemedicine appointments during the difficult times of the pandemic, as reflected by a significant squared coherence peak and phase angle in the spectrum, mirroring a significant jointly cyclical variation between outpatients’ and telemedicine activity corresponding with the COVID-19 pandemic cycle. Importantly, our data revealed that the total number of patients under our Unit’s care remained consistent over the past 2 years. This suggests that telemedicine can facilitate continuity of care for these vulnerable and aging patients, especially during challenging periods when accessing outpatient clinics becomes difficult. Telemedicine activity increased in parallel with the severity of the pandemic, as shown by the positive association between telemedicine activity and the severity of the COVID-19 pandemic in Italy. Indeed, during the first two waves of the pandemic, the rise in telemedicine appointments immediately followed the pandemic curve. These data showed how fast the Cognitive Disorders Unit could propose alternative treatments’ modality to patients and how patients positively responded to these offers. During the peaks of contagion, our Institute restricted access to outpatient clinics, but the Cognitive Disorders Unit remained open to patients throughout the pandemic. Patients were consistently provided with the option to choose between in-person and telemedicine appointments. Between the pandemic waves, patients notably preferred returning to the hospital, continuing their treatments in person at the outpatient clinic. These findings demonstrate that even during the pandemic, every patient was able to continue their treatment plan, and patients adapted effectively to the new system, ensuring a consistent continuity of care.

Interestingly, during the third wave of the pandemic in Italy, patients anticipated the increase in contagions. They made the decision, as soon as the pandemic curve began to rise again, to transition back to telemedicine appointments. This data indicates that telemedicine has become a natural choice for patients when deciding the modality of their visits (23). Nowadays, telemedicine should not be viewed merely as a replacement for outpatient visits; rather, it should be integrated into regular clinical practice, helping to provide a continuity of care and protection for vulnerable patients (7). In the patients’ and healthcare professionals’ minds, telemedicine was often perceived as an alternative or substitution of in-person appointments. Such dichotomie might create barriers in the clinical practice and might lead patients to mistrust this system. Telemedicine should be integrated as a supplementary element in clinical practice. The intensity, timing and specificity of the use of telemedicine should be personalized according to the patients diagnosis and condition. In the case of neuropsychological assistance of patients with dementia, our study suggests that telemedicine can be integrated with outpatients clinic activity. Study limitations lie in the fact that we did not investigate patients’ and caregivers’ satisfaction of the telemedicine services. Future studies should incorporate satisfactory questionnaires for both patients and caregivers, assessing the quality of neuropsychological services delivered via telemedicine and evaluating the usability of such services, including the telehealth technology.

Many efforts remain to be achieved by the healthcare systems to recognize this modality of treatment as such and implement reimbursements and payments for patients (23). Moreover, more studies are needed to define the best use of telemedicine according to the pathology being addressed. Our study showed how patients positively adhered to remote treatments for cognitive disorders. The manner in which patients transitioned back to outpatient clinic visits implies that telemedicine could be seamlessly integrated into patients’ routine care alongside in-person visits, ensuring comprehensive continuity of care. However, it’s essential to acknowledge that telemedicine might not be suitable for all conditions and there is still the need to define its best application.

## Conclusion

Our study suggests that telemedicine might constitute an effective tool to promote continuity of care for patients with dementia during the pandemic. We showed that in a short period of time, patients fully adopted this modality of treatment, switching between telemedicine and outpatients’ clinic depending on the pandemic situation. To facilitate healthcare systems in providing financial support to clinics and patients for promoting telemedicine, further studies are imperative. These studies should evaluate the amount of energy saving, the social contribution and the improvements in quality of life of patients and caregivers that might be correlated to the use of telemedicine.

## Data availability statement

The original contributions presented in the study are included in the article/supplementary material, further inquiries can be directed to the corresponding author.

## Ethics statement

The studies involving humans were approved by Ethics committee of the San Raffaele Hospital. The studies were conducted in accordance with the local legislation and institutional requirements. The participants provided their written informed consent to participate in this study.

## Author contributions

DE: Formal analysis, Writing – original draft. EH: Writing – original draft, Writing – review & editing. PR: Data curation, Writing – review & editing. AZ: Project administration, Writing – review & editing. LB: Writing – review & editing. PC: Writing – review & editing. JP: Writing – review & editing. AT: Writing – review & editing. SI: Conceptualization, Data curation, Investigation, Writing – review & editing. FA: Conceptualization, Data curation, Investigation, Methodology, Resources, Writing – review & editing.
